# Histogram equalization using a selective filter

**DOI:** 10.1007/s00371-022-02723-8

**Published:** 2022-11-29

**Authors:** Roberto M. Dyke, Kai Hormann

**Affiliations:** Faculty of Informatics, Università della Svizzera italiana, Via Buffi 13, 6900 Lugano, Switzerland

**Keywords:** Image enhancement, Dequantization, Histogram equalization, Histogram matching

## Abstract

Many popular modern image processing software packages implement a naïve form of histogram equalization. This implementation is known to produce histograms that are not truly uniform. While exact histogram equalization techniques exist, these may produce undesirable artifacts in some scenarios. In this paper we consider the link between the established continuous theory for global histogram equalization and its discrete implementation, and we formulate a novel histogram equalization technique that builds upon and considerably improves the naïve approach. We show that we can linearly interpolate the cumulative distribution of a low-bit image by approximately dequantizing its intensities using a selective box filter. This helps to distribute the intensities more evenly. The proposed algorithm is subsequently evaluated and compared with existing works in the literature. We find that the method is capable of producing an equalized histogram that has a high entropy, while distances between similar intensities are preserved. The described approach has implications on several related image processing problems, e.g., edge detection.

## Introduction

Histogram modification techniques are commonly used to enhance visual aspects of an image, such as contrast or continuity. In computer imaging systems, global histogram equalization may be applied to perceptually amplify high-frequency spatial information (e.g., edges and corners), while reducing the presence of low frequencies [1]. This is particularly useful in systems that require human–computer interaction where a user must make a decision based on the observed data.

**Fig. 1:**
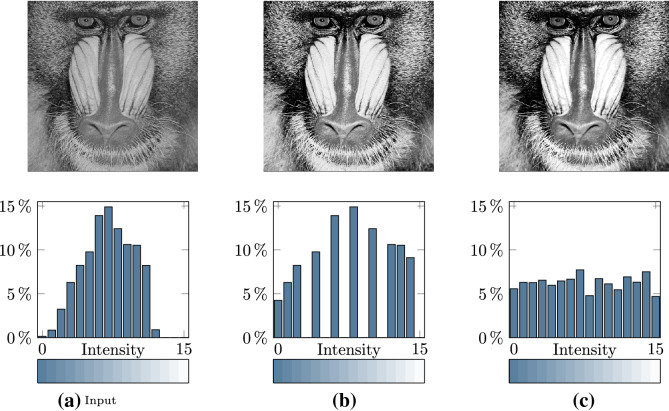
An example of histogram equalization on **a** a 4-bit image using **b** a conventional histogram equalization approach [2], and **c** the proposed method

The goal of histogram equalization is to modify the pixel intensities of an image to produce a histogram that is as uniform as possible. In information theory, this corresponds to the maximum achievable entropy. Popular photo editing software, Adobe Photoshop and GIMP (see v2.10 gimpoperationequalize.c), implement relatively naïve histogram equalization procedures that are similar to the techniques described by [2, 3]. These implementations are understood to potentially form sparse histograms [4, 5]. This is illustrated in Fig. 1, where the proposed method produces a histogram that closely resembles a fully-equalized histogram.

Commonly, for global histogram matching the *cumulative distribution function* (CDF) is used as a transfer function. The CDF of a digital image is piecewise constant (i.e., a step function). In this paper, we consider a novel formulation for the CDF in the discrete setting that produces a piecewise linear function. We consider the resulting CDF to be more faithful to the image before quantization, while increasing the problem complexity negligibly. Alone, however, this does not help address the problem of sparsity as quantized intensities map to the same value.

To redistribute intensities appropriately, we consider recovering upscaled intensities by slightly modifying a given quantized pixel’s intensity by averaging neighbouring pixels that have a similar intensity. If the neighbourhood and permitted dissimilarity is sufficiently small, this leads to only a subtle distortion of the pixelwise intensities. Through our evaluation, we find that the described approach improves the quality of the resulting histogram. Two parameters are used to control the effect of this technique on the histogram, and enable the preservation of relations between pixels of the same quantized intensity. Applying the most restrictive parameters causes the proposed method to achieve parity with commonly used approaches [2, 3].

In this paper, we suggest that through moderate local pixelwise modification of the original image artifacts, caused by intensity quantization, in the histogram space may be reduced.

The technical contributions of this work may be summarized as follows:

- An adaptive kernel-based method that seeks to address the issue of histogram sparsity for down-stream applications (e.g., histogram equalization and histogram matching).
- A thorough evaluation of the proposed technique, including practical parameter selection experiments and comparisons with various pertinent approaches.

## Related work

Many approaches to contrast enhancement have been proposed over the last half-century [6–8], leading to a wide range of histogram equalization techniques [9]. This section provides an overview of the relevant literature that addresses aspects of this problem.

### Histogram equalization

Histogram equalization is a commonly used enhancement technique to increase the visual contrast of an image in applications, such as medical imaging, robotics, and astronomy. This is particularly useful in systems that require human–computer interaction where a user must make a decision based on an image. In computer vision systems, histogram equalization may be applied locally to enhance high-frequency spatial information (e.g., edges and corners), while reducing the presence of low frequencies [1]. The technique may also be used in image coding.

Given a greyscale image, the goal is to compute a transformation that, when applied to the gray values of the original image, produces a uniform distribution of the intensity values.

The origins of the now pervasive global histogram equalization procedure [3] are obscure; however, as with other image processing algorithms [10], the techniques used for histogram equalization are highly associated with techniques from statistics [11]. In its simplest form, the method follows the description by [2]. Ketcham et al. [12] propose a technique that uses a two-dimensional sliding window over an image’s spatial domain. Histogram equalization is performed within the small window to compute the equalized intensity value of either the central pixel (or a group of central pixels). Subsequently, variations of this technique known as ‘adaptive histogram equalization’ have been considered [1, 13, 14]. Adaptive histogram equalization techniques improve contrast locally but cause the contrast enhancement to no longer be a global transformation. Notably, [15] introduce bi-histogram equalization. Unlike adaptive histogram equalization, where multiple histograms are constructed based on the spatial relationship of pixels, bi-histogram equalization constructs multiple histograms based on the similarity of intensities. An image is partitioned by its mean intensity, then histogram equalization is independently applied to both parts. This technique aims to preserve the mean brightness of an image, but may not obtain the maximum entropy when the number of pixels assigned to each partition differs. Wang et al. [16] address this by partitioning the *probability mass function* (PMF) of the image into equal areas (i.e., using the median intensity). Many works have sought to enhance the contrast in an image locally while imposing brightness preserving constraints [17–19]. The use of piecewise-linear representations for histogram modification are well-known [5]. This representation has been applied to histogram equalization, where it serves as an approximation of the CDF [20, 21]. However, current approaches in the literature do not address the problem of sparsity in the resulting histogram.

Hall [4] identifies that digitized images do not produce a uniform histogram when using the naïve histogram equalization technique. Rather than constructing a typical CDF, a family of techniques seek to address this problem by determining a strict order for pixels based on their intensities. Given an ordering, pixels are then divided into *l* evenly spaced bins that correspond to a pixel intensity. These techniques are capable of maximizing the entropy of the resulting equalized histogram; however, the general approach is not infallible. The principle challenge is deciding how to appropriately handle ties—where two pixels have the same intensity—without this, a strict ordering is not possible. A series of works [22–24] apply a series of low-pass (blurring) filters of varying neighbourhood sizes on an input image to establish an order for pixels of the same intensity. While this approach does rely on spatial information, it can cause undesirable blurring along edges and at corners. An additional problem is the enhancement of noise in an image. Nikolova et al. [25] attempt to dequantize an image approximately by using a variational-based optimization approach on the image’s graph structure that may help with some quantization noise. The intensity of pixels in the dequantized image tends to be unique, so a strict ordering for histogram specification may be applied. Similarly, the proposed method performs dequantization as an intermediary step, and can therefore be incorporated into a similar pixel ordering framework.

While strict ordering methods produce perfectly flat histograms, such techniques degenerate in scenarios where the original image contains sparse intensities.

### Dequantization

Key to the proposed method is the conversion of integer-based pixelwise intensities into a floating-point representation that approximates the original pixel’s intensity before quantization. The problem of dequantization has been examined in works mostly related to bit-depth expansion [26–29] and inverse tone mapping—or *high dynamic range* (HDR) reconstruction—[30] both via optimization [31–33] and deep learning [34–36] techniques. Broadly, the goal of these methods is to—given a quantized image—recover the original (dequantized) image while suppressing perceptual artifacts (e.g., noise, false contours, half-toning and edge preservation). Many notable works have investigated these problems: false contours [26, 37, 38], half-toning [39, 40], and preserving edges [41]. These solutions could theoretically help to address our dequantization problem; however, they introduce unnecessary assumptions about the content of an image that may increase the dequantization error to reduce visual artifacts, rather than necessarily ensuring the quality of the histogram.

Other works consider the problem of recovering an HDR image from a low dynamic range source. Recent techniques in this area often employ deep learning frameworks to address related problems, e.g., inverse half-toning [42, 43], removing false contour artifacts [44], and exposure correction of an image in challenging lighting scenarios [36, 45]. In practice, these methods generally suffer from the problem of data scarcity, which is not typically a problem for hand-engineered approaches.

The closest related work to the proposed kernel is that of [33]. The authors apply a sparse adaptive filtering technique to remove artifacts caused by intensity quantization. To preserve edges, the smoothing filter is only applied when the intensities of the neighbouring pixels are within a sufficient delta of the central pixel that is determined using [32]—a technique for inverse tone mapping. A follow-up work formulated a procedure for selecting optimal parameters [46]. We illustrate that this approach is very cautious about where filtering is applied, greater locality could be achieved using smoothing filters that preserve edges (e.g., [47]).

Chen et al. [32] construct a continuous representation by fitting a polynomial equation to the intensity transformation function. For greater accuracy, rather than using a high-order polynomial, the authors propose to arbitrarily split the intensity space and approximate piecewise polynomials.

## Background

Without loss of generalization, a monochrome *image* can be seen as a piecewise continuous bivariate function $f:\Omega \rightarrow I$ that assigns to any point (*x*, *y*) from the *domain* $\Omega =[0,1]\times [0,1]$ an *intensity* *f*(*x*, *y*) in the range $I=[0,1]$. A *digital image* ${\textbf{I}}$ is a discrete representation of *f*, with the domain partitioned into $m\times n$ *pixels* and the intensity quantized to the discrete range $L=\{0,1,\dots ,l-1\}$. Usually, ${\textbf{I}}$ is given in terms of a matrix of values $I_{i,j}\in L$, for $i=0,1,\dots ,m-1$ and $j=0,1,\dots ,n-1$, where $I_{i,j}$ is the discrete intensity of the (*i*, *j*)-th pixel.

To convert a given image *f* into a digital image ${\textbf{I}}$, the pixel intensities can be determined, for example, by sampling *f* at the pixel centres,

$$\begin{aligned} f_{i,j} = f \bigl ( (i+\tfrac{1}{2})\Delta _x, (j+\tfrac{1}{2})\Delta _y \bigr ), \end{aligned}$$

where $\Delta _x=1/m$ and $\Delta _y=1/n$, or by averaging *f* over each pixel,

$$\begin{aligned} f_{i,j} = \int _{j\Delta _y}^{(j+1)\Delta _y} \int _{i\Delta _x}^{(i+1)\Delta _x} f(x,y)\,\textrm{d}x\,\textrm{d}y, \end{aligned}$$

and then quantizing $f_{i,j}$ by setting

1 $$\begin{aligned} I_{i,j} = {{\,\textrm{round}\,}}\bigl ( (l-1) f_{i,j} \bigr ). \end{aligned}$$

In this paper, we consider the rounding operator with the “round half up” tie-breaking rule and hence assume that ${{\,\textrm{round}\,}}(x)=\lfloor x+\tfrac{1}{2} \rfloor$.

Vice versa, a digital image ${\textbf{I}}$ can be seen as a bivariate image function *f* with constant intensity over the rectangles covered by each pixel, that is,

$$\begin{aligned} f(x,y) = \Delta _l I_{i,j}, \end{aligned}$$

where $$\Delta _l=1/(l-1)$$ and

$$\begin{aligned} i = {\left\{ \begin{array}{ll} \lfloor mx \rfloor , &{} x\in [0,1),\\ m-1, &{} x=1, \end{array}\right. } \quad j = {\left\{ \begin{array}{ll} \lfloor ny \rfloor , &{} y\in [0,1),\\ n-1, &{} y=1. \end{array}\right. } \end{aligned}$$

### Histogram equalization

**Fig. 2:**
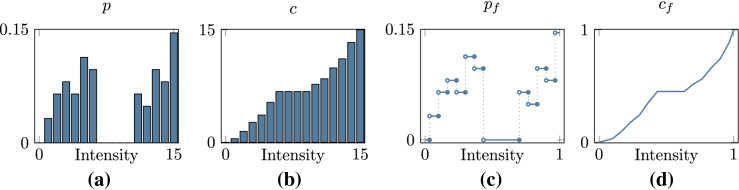
Histograms of the typical **a** PMF, and **b** (discrete) CDF computed for histogram equalization. The corresponding **c** PDF, **d** (continuous) CDF produced by the new method without upscaling the intensities

Let us first consider the continuous setting. Denoting by $p_f:I\rightarrow [0,1]$ the *probability density function* (PDF) of an image *f*, it is well known [3] that transforming the intensities of *f* with the CDF $c_f(t)=\int _0^t p_f(s)\,\textrm{d}s$ gives an image $f'=c_f\circ f$ with uniform PDF $p_{f'}\equiv 1$.

In essence, discrete methods seek to emulate this process. For a digital image ${\textbf{I}}$, this *histogram equalization* procedure is usually discretized as follows [2].

1. *Construct a histogram of the pixelwise intensities of an image.* We first determine the probability of a pixel in ${\textbf{I}}$ to have a specific intensity,
  2 $$\begin{aligned} p(k) = \frac{h(k)}{mn}, \qquad k\in L, \end{aligned}$$
  where $h(k)=\#\{(i,j):I_{i,j}=k\}$ is the number of pixels in ${\textbf{I}}$ with discrete intensity *k*, forming the PMF $p:L\rightarrow [0,1]$ (shown in Fig. 2a).
2. *Compute the cumulative distribution function.* As illustrated in Fig. 2b, we then accumulate and quantize these probabilities to produce the *discrete* CDF $c:L\rightarrow L$,
  3 $$\begin{aligned} c(k) = {{\,\textrm{round}\,}}\biggl ( (l-1) \sum _{i=0}^k p(i) \biggr ), \qquad k\in L. \end{aligned}$$
  By construction, $c(l-1)=l-1$. When, $c(0) > 0$, *c* may be scaled such that $c(0)=0$ [3].
3. *Back project intensities using the cumulative distribution function.* Finally the pixel intensities of the processed image ${\textbf{I}}^*$ are set to $I^*_{i,j}=c(I_{i,j})$, for $i=0,1,\dots ,m-1$ and $j=0,1,\dots ,n-1$. The resulting image has an intensity histogram where the bins are approximately equalized.

We consider this as a baseline approach to histogram equalization. It may be noted that other descriptions [3, 6, 48] scale the output of the CDF to ensure that the output value range (e.g., for an 8-bit image) populates the first and last histogram bins, at 0 and 255. The fundamental problem with such approaches is that, after transformation, the resulting PMF of the equalized image is often sparse [4] (see Fig. 1a). This means that the available discrete intensity values are not fully utilized. With the proposed method, this problem is assuaged.

## The new method

In order to improve the classical approach to histogram equalization of digital images, we propose to adopt the continuous setting more carefully. To this end, we assume that the given digital image ${\textbf{I}}$ is the discrete representation of some image *f*. According to (1), each discrete intensity $k\in L$ represents some continuous intensity $t\in I$ with

4 $$\begin{aligned} (k-\tfrac{1}{2}) \Delta _l \le t < (k+\tfrac{1}{2}) \Delta _l. \end{aligned}$$

Under the assumption that the PDF of *f* is *uniform* for all intensities that get quantized to the same discrete intensity, we conclude that $p_f:I\rightarrow I$ is a piecewise constant function with

$$\begin{aligned} p_f(t) = \frac{p(k)}{\Delta _l} \cdot {\left\{ \begin{array}{ll} 1, &{} k\in \{1,2,\dots ,l-2\},\\ 2, &{} k\in \{0,l-1\}, \end{array}\right. } \end{aligned}$$

where $k={{\,\textrm{round}\,}}((l-1)t)$ and *p*(*k*) as in (2), illustrated in Fig. 2c. Note that the factor 2 owes to the fact that the intervals $\bigl [0,\tfrac{1}{2}\Delta _l\bigr )$ and $\bigl [1-\tfrac{1}{2}\Delta _l,1\bigr ]$ of intensities that are quantized to the discrete intensities $k=0$ and $k=l-1$, respectively, are half as big as the other intervals. Consequently, the CDF of *f*, $c_f:I\rightarrow I$, is a piecewise linear function over the partition $\bigl [0,\tfrac{1}{2}\Delta _l,\tfrac{3}{2}\Delta _l,\dots ,\tfrac{2l-3}{2}\Delta _l,1\bigr ]$ of *I* with $c_f(0)=0$, $c_f(1)=1$, and

$$\begin{aligned} c_f \bigl ( (k+\tfrac{1}{2}) \Delta _l \bigr ) = \sum _{i=0}^k p(i), \qquad k=0,1,\dots ,l-2, \end{aligned}$$

as shown in Fig. 2d.

Using this model, any discrete intensity $k\in L$ is first converted to a continuous value, then transformed by $c_f$, and finally quantized back to *L*, that is,

5 $$\begin{aligned} {\tilde{c}}(k) = {{\,\textrm{round}\,}}\bigl ( (l-1) c_f(\Delta _l k) \bigr ). \end{aligned}$$

Since

$$\begin{aligned} c_f(\Delta _l k)&= \frac{c_f\bigl ((k-\tfrac{1}{2})\Delta _l\bigr ) + c_f\bigl ((k+\tfrac{1}{2})\Delta _l\bigr )}{2}\\&= \sum _{i=0}^{k-1} p(i) + \frac{1}{2} p(k), \end{aligned}$$

for $k\in \{1,2,\dots ,l-2\}$, this turns out to be very similar to the classical approach (cf. (3)), yielding almost identical processed images.

**Fig. 3:**
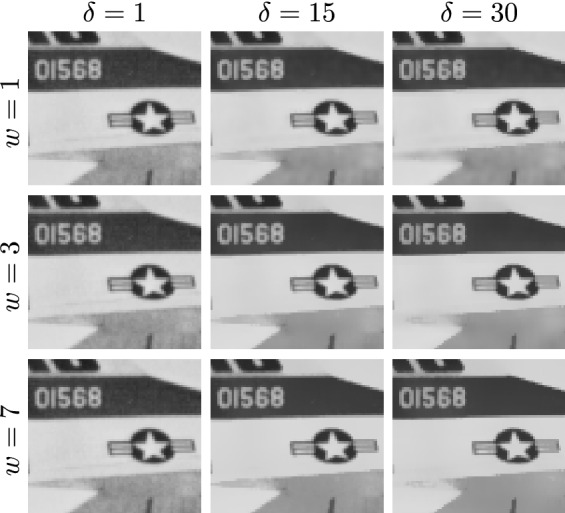
Given a portion of the image in Fig. 6a, we show the effect of applying our proposed filtering without performing any upscaling or equalization. In each row, the parameter *w* is varied, while in each column $\delta$ is varied. Looking at $\delta$ in isolation, we notice that key noise gradually disappears, while strong edges are preserved

**Fig. 4:**
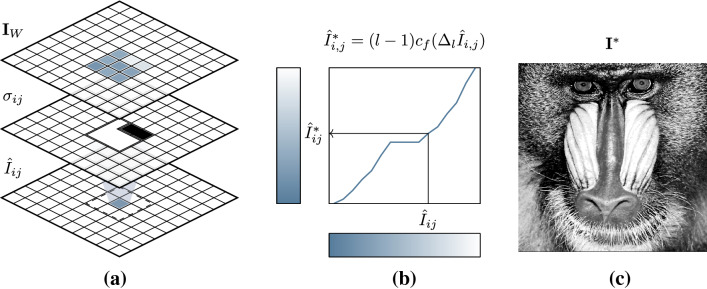
An illustration of the key steps of the proposed algorithm. **a** Given an image ${\textbf{I}}$, at each pixel location (*i*, *j*) a neighbourhood of discrete pixel intensities in a window ${\textbf{I}}_W$ are sampled. Neighbouring pixels of significantly dissimilar intensity are filtered by $\sigma _{i,j}$, and the average of the remaining pixels is used to compute the dequantized pixel intensity ${\hat{I}}_{i,j}$. **b** an augmented cumulative distribution $c_f$ is used as a continuous look-up table for equalization. **c** A discrete equalized image is recovered, where ${\textbf{I}}^*_{i,j} ={{\,\textrm{round}\,}}({\hat{I}}^*_{i,j})$

### Intensity upscaling

The crucial next step is to further reason about the intensity $t\in I$ that is represented by the discrete intensity $k=I_{i,j}\in L$ of the (*i*, *j*)-th pixel of ${\textbf{I}}$. So far, we assumed *t* to be the midpoint $t=\Delta _l k$ of the interval in (4), which is a reasonable guess in the absence of further information, but we can do better, if we take the intensities of neighbouring pixels into account. To this end, recall that ${\textbf{I}}$ is the discrete representation of some image *f*, which is assumed to be piecewise continuous. In a first step, we therefore identify all those neighbouring pixels with an intensity similar to $I_{i,j}$ by defining the binary *similarity mask*

$$\begin{aligned} \sigma _{i,j}(u,v) = {\left\{ \begin{array}{ll} 1, &{} \text {if}~ |I_{i+u,j+v}-I_{i,j}| \le \delta ,\\ 0, &{} \text {otherwise,} \end{array}\right. } \end{aligned}$$

for some *similarity threshold* $\delta$ and a square neighbourhood window *W* of radius *w*, that is, for $(u,v)\in W=\{(x,y)\in {\mathbb {Z}}^2:|x|,|y|\le w\}$. Nearby pixels with $\sigma _{i,j}(u,v)=1$ are now assumed to correspond to the same continuous piece of *f* and their intensities can be used to reconstruct this piece locally. In the simplest setting, we may fit a constant function to these intensities in the least-squares sense and take its value as a better estimate of *t*. A straightforward calculation reveals that this amounts to applying a *selective box filter*, which simply averages neighbouring similar intensities,

$$\begin{aligned} {\hat{I}}_{i,j} = \frac{1}{\# W'} \sum _{(u,v)\in W'} I_{i+u,j+v}, \end{aligned}$$

where $W'=\{(x,y)\in W:\sigma (x,y)=1\}$, and provides the continuous intensity estimate $t=\Delta _l{\hat{I}}_{i,j}$. It remains to transform this value by $c_f$ and to quantize the result to *L*, so as to get the discrete intensity of the (*i*, *j*)-th pixel in ${\textbf{I}}^*$, that is, to set $I^*_{i,j}={\tilde{c}}({\hat{I}}_{i,j})$ for $i=0,1,\dots ,m-1$ and $j=0,1,\dots ,n-1$, with ${\tilde{c}}$ defined as in (5), but more generally for any real-valued argument in $[0,l-1]$.

The influence of the parameters *w* and $\delta$ used just to filter an image is illustrated in Fig. 3.

### Implementation

Figure 4 gives a visual overview of the proposed method, which comprises of two key parts: (1) a dequantization procedure; and (2) a piecewise linear CDF.
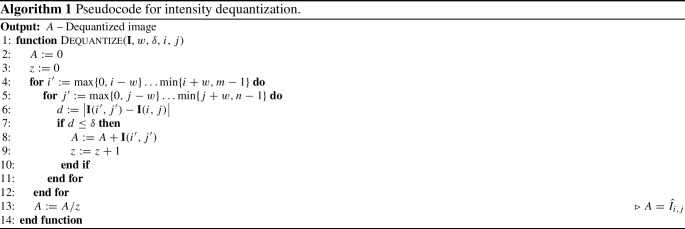


Algorithm 1 describes how a pixel is dequantized with respect to its neighbours programmatically. As we demonstrate empirically, the proposed technique performs particularly well on images that have smooth intensity gradients; however, this may be replaced with an application-specific technique.
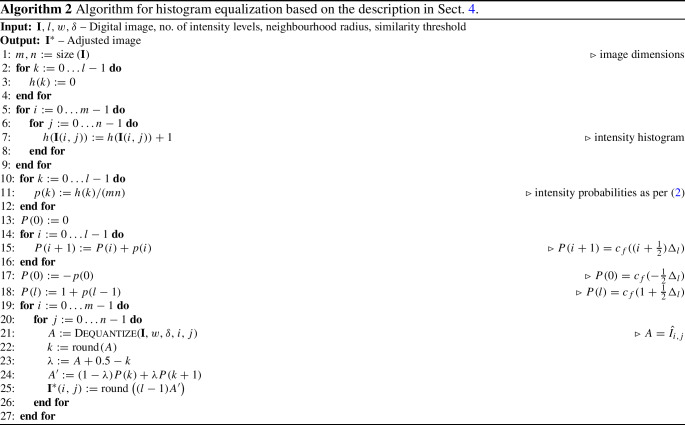


The pseudocode for histogram equalization is given in Algorithm 2. By constructing the CDF using the original discretized intensities, the proposed approach avoids increasing the space complexity of the CDF, which a perturbed real-value image would require. N.B.: lines 17 & 18 are a necessary modification for the linear interpolation used on line 24.

The proposed method has a complexity of ${\mathcal {O}}(w^2mn)$. In practice, we find that the optimal value for *w* is likely to be small; therefore, *w* only has a small influence on the algorithm’s speed.

## Evaluation

Qualitative results of experiments involving the methods described in the following section are discussed. Further quantitative evaluation of the proposed method is included in “Appendix A”.

### Benchmark methods

For the evaluation, a range of representative techniques that may be used for histogram equalization were selected. Each method was implemented in MATLAB. While execution times are reported, it is expected that the runtime of each method could be greatly reduced in a low-level language.

#### Naïve equalization (baseline)

Implements the discrete histogram equalization procedure as described in Sect. 3.1.

#### Naïve scaling (baseline)

For intensity upscaling tasks, intensities are uniformly scaled and then rounded.

#### Bi-linear interpolation

The spatial resolution of the image is increased by doubling its dimensions using bi-linear interpolation. Naïve histogram equalization is then applied to the interpolated image before re-scaling the image back to its original dimensions.

#### Coltuc et al.  [23]

**Fig. 5:**
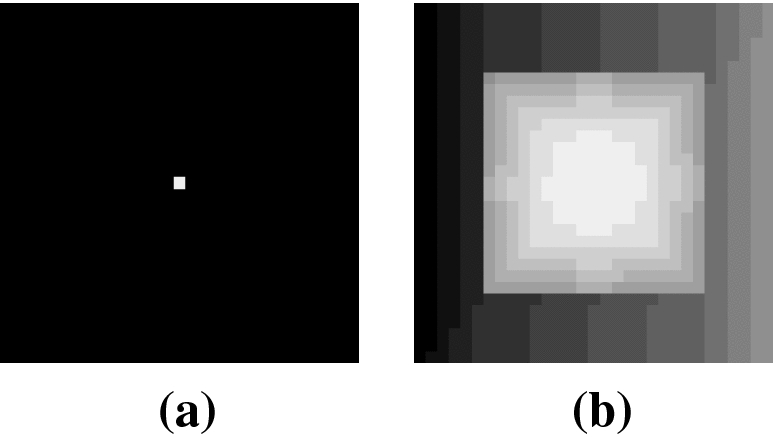
An example of **a** a synthetic 31-by-31 pixel 4-bit image and **b** after exact histogram equalization by a specification method [23]. Due to the assumption that there exists an appropriate order, the method produces undesirable results when intensities are sparse. The other approaches, evaluated later, produce the same results as naïve histogram equalization, which looks like (**a**)

Given an image, pixels are assigned an order based on their intensity value. To determine an order between pixels with the same intensity, the tied pixels are blurred with respect to their neighbours. The ties are then sub-ordered by their new intensity. This tiebreaker process may be repeated using successively larger blur windows, until all ties are resolved. While uncommon in real-world images, when intensities in the quantized image are particularly sparse, this can lead to significant artifacts. A synthetic example of this problem is shown in Fig. 5.

#### Song et al. [33]

The method is designed for image dequantization. A sparse kernel is used for efficiently smoothing false contours. For histogram equalization tasks, we still follow Algorithm 2, replacing the proposed dequantization method (Algorithm 1) with the authors’ described algorithm [33].

**Fig. 6:**
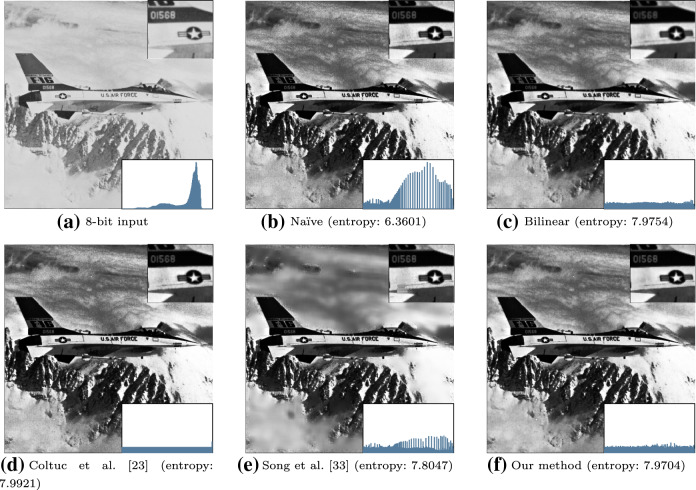
Qualitative results of the benchmarked histogram equalization methods on **a** a real 8-bit image. The intensity histograms given all share the same limits. **b** Retains natural image noise, while the equalization is sub-optimal. **c** Applies a slight blur to the entire image. **d** Perfectly equalizes the histogram, while enhancing noise. **e** The performance of [33] is the same as the naïve method in high contrast areas. **f** Softens noise in the image, while preserving details such as the text and star on the side of the plane ($w=1$ & $\delta =4$)

**Fig. 7:**
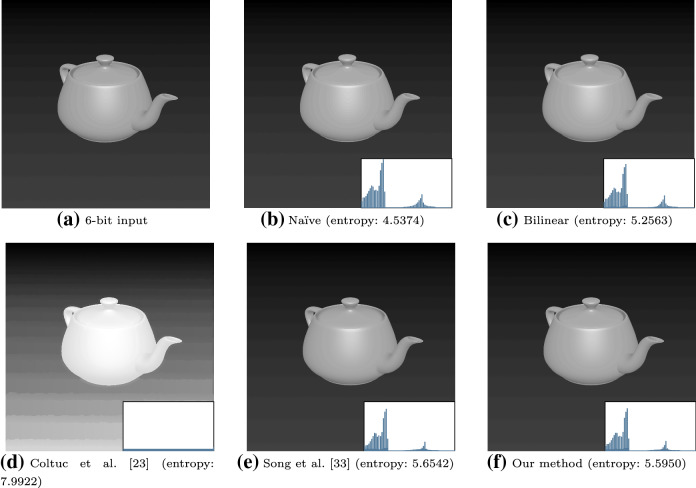
Qualitative results of the benchmarked histogram equalization methods on a synthetic 6-bit image upscaled to 8 bits. [23] enhances artifacts present in the image

**Fig. 8:**
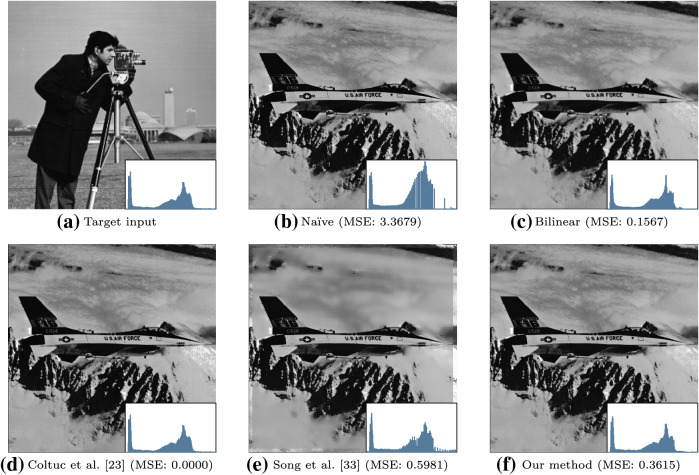
An example of histogram matching of the overexposed image in Fig. 6a and the histogram of (**a**). The MSE between the adjusted and target histogram is given below each image (note that the MSE has been scaled by $\times 10^5$). As in Fig. 6, similar perceptual artifacts are observed

**Fig. 9:**
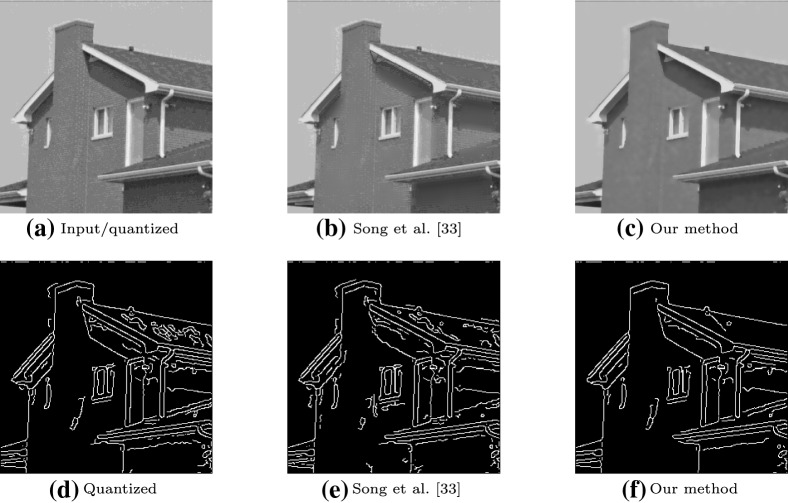
An example of edge preservation. **a** The 4-bit image used as input. Columns **d**–**f** show the result of applying the Canny edge detector. The result of performing edge detection on the quantized image is shown in (**d**). **b**, **c** The result of dequantizing the image using Song et al. [33] and our method before performing edge detection. **d**, **e** Contain undesirable noise caused by artifacts from the initial quantization of (**a**), which are suppressed in (**f**) by the proposed method

### Benchmark datasets

Two sets of images were collected, one of noiseless synthetic images, as well as a set of images captured by typical digital cameras that contain natural noise. For quantitative experiments (“Appendix A”), the original images were treated as ground truths, while a quantized version of each image was used as input.

#### Synthetic dataset

Synthetic 3D objects were rendered such that no noise was captured by a virtual camera. The shapes were textureless and conform to the assumption that an image comprises of piecewise-linear patches. These were primarily used for intensity upscaling experiments, described in “Appendix A.3”.

#### Real dataset

Illustrative images presented in this section were obtained from the USC-SIPI Image Database (https://sipi.usc.edu/database/).

For histogram equalization experiments, presented in “Appendix A.4”, a moderately sized database of 1449 real images was collected using the Flickr API. The following keywords were used to collect a range of real images: *car, Cuba, pedestrian, tiles,* and *windmill*.

### Histogram equalization

Exemplar results that are representative of each method are shown in Figs. 6 and 7. For each processed image, the entropy is reported. Entropy can be viewed as a measure of uniformity of the distribution of a PDF. An appropriate measure is *Shannon’s entropy*, which is defined as

$$\begin{aligned} H({\textbf{I}}^*) = \sum _{i=1}^{l} \left[ p(i)\log _2\frac{1}{p(i)} \right] . \end{aligned}$$

The bounds are $0 \le H({\textbf{I}}^*) \le \log _2l$, where $\log _2l$ is the maximum entropy, which represents a uniform PDF. Further results are included in “Appendix A.4”.

In Figs. 6 and 7, we find that [23] obtains the greatest entropy. However, in Fig. 7, [23] enhances the presence of false contours. The bilinear interpolation method achieves comparable results to the proposed technique; however, due to the lack of edge-preservation, it smooths the entire image slightly. We emphasize that the selectivity of the proposed smoothing filter allows it to controllably preserve edges.

The extension to histogram matching for each method is relatively trivial and therefore omitted. Qualitative results are presented in Fig. 8.

### Edge preservation

Consider a surface that exhibits a piecewise linear signal $\varvec{x}$ with sharp discontinuities where edges are present (e.g., a step function). Simply applying an averaging filter to a quantized signal can smooth out quantization errors with intervals, but this will also smooth the boundaries between separate intervals in $\varvec{x}$. This is undesirable. A simple yet effective way to preserve the piecewise quality of $\varvec{x}$ when filtering is to exclude highly dissimilar neighbouring values, as these are more likely to be part of a separate interval.

We demonstrate the ability of our method to correctly preserve edges by applying Canny edge detection [49] to an image that we dequantize in Fig. 9. While, to the naked eye, the dequantized image (on the top row) of our method (where $w=2$ & $\delta =1$) appears to contain false contours, the results demonstrate that these are sufficiently smoothed for the purposes of edge detection. The proposed method produces a binarized image with less noise than Song et al. [33], which preserves false contours near edges, because the method strictly does not apply dequantization in locations where the intensity of one or more neighbouring pixels is greater than a determined threshold.

## Discussion

While the focus of this paper has been histogram equalization, the applications of the proposed technique extend beyond this scope. Our method can be easily applied to histogram matching and adaptive histogram equalization. Also, similar to [23], using the proposed upsampling procedure to determine an order of the intensities could enhance the results for exact histogram equalization.

The preceding discussion in Sect. 4 can also be adapted for the problem of bi-histogram equalization. Many previous works (e.g., [15–17, 50, 51]) select an intensity to partition an image into two or more parts, let us call this boundary value *b*. In the finite setting, image quantization introduces errors that cause pixels to be assigned to the incorrect partition with respect to *b*. Therefore, the dequantized image should be used to accurately determine the partitioning. An upper PMF and lower PMF can then be constructed as previously described.

A key limitation of this work is the procedure used for image upscaling. In our experiments, Algorithm 1 helped to accurately dequantize synthetic images. However, for real images, it was only shown to improve the histogram quality. It is possible that Algorithm 1 may be further improved by applying a Gaussian weighting to the contribution of neighbouring pixels, or by replacing this part entirely with a data-driven technique.

A further consideration is that it is possible that a pixel that is darker than another pixel in the original image could be switched—such that the darker pixel is brighter than the other pixel in the adjusted image. The occurrence of these are bounded by $\delta$, as $\delta$ becomes smaller, the impact of this reduces.

## Conclusions

In this paper, we consider the problem of recovering high-quality histograms from low bit-depth images. Through fundamental reasoning about what a pixel and its neighbours represent, a simple yet effective technique is proposed to transform discrete pixel intensities into continuous values. Consequentially, the data better reflects the continuous theory for histogram equalization that is commonly followed in the discrete setting.

Only two parameters (*w* & $\delta$) are used to finely balance the level of intensity error and entropy, while [23] offers no parameters, and [33] requires many that are complex to tune. Parameter selection experiments conducted on our method revealed that, for real images, the optimal parameters tend to be small values. In specific applications replacing the technique used for dequantization with a bespoke algorithm may further enhance the results.

Implementations for GIMP (in Python) and Paint.NET (in C#) are available.

## Figures and Tables

**Fig. 10:**
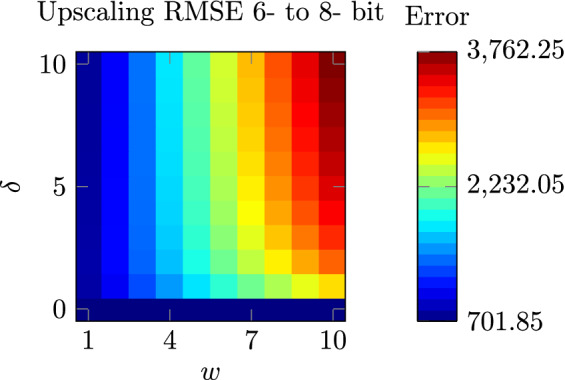
The average pixelwise RMSE of a parameter selection experiment in which *w* & $\delta$ were varied, for the task of image upscaling, to determine the optimal parameters $w=1$ & $\delta =0$. When $\delta =0$, varying *w* has no effect, the resulting error was 701.8471

**Fig. 11:**
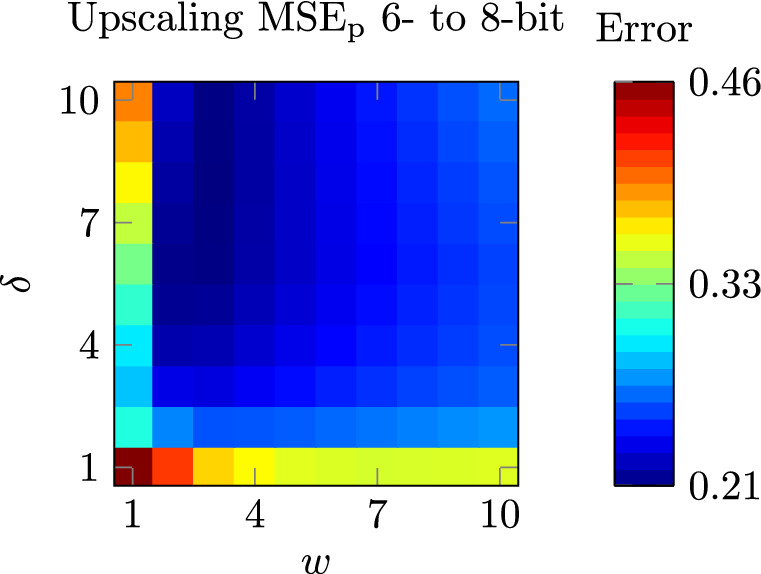
The average histogram error ($\text {MSE}_\text {p}$) of a parameter selection experiment in which *w* & $\delta$ were varied, for the task of image upscaling. When $\delta =0$, varying *w* has no effect, the resulting error was 1.5090

## Data Availability

Data sharing is not applicable to this article as no datasets were generated or analysed during the current study.

## Additional experiments

### Quantitative measures

Both *root mean square error* (RMSE) and *peak signal to noise ratio* (PSNR) are used to quantify the difference between each up-sampled image ${\textbf{I}}^*$ and the corresponding ground truth image ${\textbf{I}}^\text {gt}$. As [52] conclude, typical measures like PSNR and *structural similarity* (SSIM) are—relatively—insensitive to Gaussian blur.

Additional measures used to help determine the quality of the histogram are detailed here.

#### Mean square error of *p*

The mean sum of squared errors between the PDF of the original 8-bit image $p^\text {gt}$ and the PDF of the dequantized (e.g., upscaled from 6-bit to 8-bit) image $p^*$, i.e., 

$$\begin{aligned} \text {MSE}_\text {{p}}({\textbf{I}}^{\text {gt}};\,{\textbf{I}}^{*}) = \frac{1}{l_\text {gt}} \sum ^{l_\text {gt}}_{i=1} \left[ p^{\text {gt}}(i)-p^*(i) \right] ^{2}, \end{aligned}$$

where $p^\text {gt}$ and $p^*$ are calculated as in (2).

#### Dequantization error

The mean sum of squared errors of the gradient between the CDF of ${\textbf{I}}^\text {gt}$ and ${\textbf{I}}^*$, i.e., 

$$\begin{aligned} \text {DE}({\textbf{I}}^\text {gt};\,{\textbf{I}}^*) = \frac{1}{l_\text {gt}} \sum ^{l_\text {gt}}_{i=1} \left( \left[ c^{\text {gt}}(i)-c^*(i)\right] ' \right) ^2, \end{aligned}$$

where $c^\text {gt}$ and $c^*$ are computed using (3) for all methods. This error measure produces a larger error for methods that fail to dequantize the input image—causing a jagged appearance in the resulting CDF. Compared with the $\text {MSE}_\text {p}$ error, this measure exhibits greater invariance to shifts in illumination, provided the rate of change in illumination matches.

#### Histogram deviation

Ideally, an equalized histogram should be dense and flat. Additionally, in the transformed image, one expects pixels of the same intensity to be mapped to share the same new intensity—deviations may be considered to be errors. Histogram deviation quantifies these deviations.

For each intensity $k=0,\dots ,l_\text {gt}-1$, we find pixels of the same intensity in the original image

$$\begin{aligned} S_k= \left\{ (i,j) \in {\textbf{M}}: {\textbf{I}}_{i,j}^\text {gt} = k \right\} , \end{aligned}$$

where ${\textbf{M}}=\{0,\dots ,m-1\} \times \{0,\dots ,n-1\}$, compute the mean after equalization

$$\begin{aligned} M(k) = \frac{1}{|S_k|} \sum _{(i,j) \in S_k}^{|S_k|} {\textbf{I}}^*_{i,j}, \end{aligned}$$

and then the variance is

$$\begin{aligned} {{\,\textrm{var}\,}}(k) = \frac{1}{|S_k|} \sum _{(i,j) \in S_k}^{|S_k|} \left[ {\textbf{I}}^*_{i,j} - M(k) \right] ^2. \end{aligned}$$

Once computed for each $0 \le k < l_\text {gt}$, the mean intensity error can be measured as

$$\begin{aligned} \frac{1}{l_\text {gt}} \sum _{k=0}^{l_\text {gt}-1} {{\,\textrm{var}\,}}(k), \end{aligned}$$

and the maximum intensity error is $\max _k {{\,\textrm{var}\,}}(k)$.

### Parameter selection for upscaling

The proposed method introduces two parameters that require tuning, the window size *w* and the intensity threshold $\delta$. These parameters are used for the estimation of the original intensity value of a given pixel before quantization was applied. To determine the optimal values, a range of parameters were exhaustively tested. For this experiment, the set of real images collected from Flickr was used.

Given an 8-bit image, a 6-bit version was produced. The low bit-depth image was then dequantized by the proposed method using the given parameters. The proposed dequantization procedure (Algorithm 1) is applied to each pixel, then the intensity is requantized at the new scale, i.e., ${\textbf{I}}^*(i,j):=A/z \cdot (l_2-1)/(l_1-1)$. Finally, the error was measured between the dequantized image and the original 8-bit image in the form of the RMSE of ${\textbf{I}}^*$ and the histogram error of $p^*$.

Figure 10 shows the RMSE between ${\textbf{I}}^\text {gt}$ and ${\textbf{I}}^*$. For this database the optimal parameters were obtained when the *w* was small (i.e., $w=1$, meaning the window spanned $3 \times 3$ pixels). The optimal value of $\delta$ varies depending on what is considered to be the priority for a given dataset—histogram error or pixelwise error. Setting $\delta =0$ causes the method to be almost equivalent to the naïve scaling method; therefore, the optimal value is likely to be small (i.e., $\delta \le 5$).

Figure 11 shows the histogram error between $p^\text {gt}$ and $p^*$. The accuracy was found to improve greatly when $w>1$; however, as Fig. 10 shows, this increases the RMSE score by subtly blurring the image. As $\delta$ is increased the prominence of this undesirable blur effect is also increased.

### Intensity upscaling

For dense histogram equalization, the proposed method relies upon recovering a floating point intensity value. Ideally, the image is correctly dequantized, leading to a histogram that accurately represents that of the original (continuous) image.

Results on synthetic images are given in Table 1. Understandably, the proposed method has a slower execution time than simpler methods. However, in terms of the reported accuracy measures, it was found to out-perform the other examined methods at intensity upscaling over a range of parameters. The error manifests in the form of a slight blurring over areas with similar intensity, while the sharpness of edges is sufficiently preserved when $\delta$ remains small.

**Table 1:** Experiments on a small set of synthetic images in which number of intensities was increased from 14-bit to 16-bit

Method	RMSE	PSNR (dB)	$\text {MSE}_\text {p}$ (avg. error $\times 10^3$)	Dequantization (avg. error $\times 10^4$)	Time (s)
Naïve scaling (baseline)	0.9646	48.8424	1.0430	2.6360	**0**.**0328**
Bi-linear interpolation	1.4906	44.8040	1.0078	2.5320	0.1760
Coltuc et al. [23]	94.0358	9.1643	1.3176	6.5920	5.2415
Song et al. [33]	0.8871	**49**.**8583**	1.2555	3.1425	37.6417
Our method ($w=1$, $\delta =0$)	0.9646	48.8424	1.0430	2.6360	19.2229
Our method ($w=1$, $\delta =1$)	0.9008	49.6043	1.0122	2.5440	18.9384
Our method ($w=1$, $\delta =2$)	0.9693	48.9541	1.0092	2.5320	18.9429
Our method ($w=1$, $\delta =5$)	1.1889	47.0812	1.0078	2.5280	19.1186
Our method ($w=2$, $\delta =1$)	**0**.**8849**	49.7869	1.0094	2.5360	19.1533
Our method ($w=2$, $\delta =2$)	0.9842	48.8386	1.0046	2.5260	19.2021
Our method ($w=2$, $\delta =5$)	1.3387	46.0500	**0**.**9992**	**2**.**5060**	19.4689

Bold indicates the best performance in each column

**Table 2:** Results of experiments measuring deviation of intensities when performing histogram equalization and the average entropy of the equalized histogram

Method	Hist. deviation $\times 10^3$		Entropy
	Avg. mean	Avg. max	
Naïve equalization (baseline)	**0**.**0000**	**0**.**0000**	7.0045
Bi-linear interpolation	1.5134	9.5209	7.8493
Coltuc et al. [23]	0.0051	0.2930	**7**.**9922**
Song et al. [33]	0.4465	2.9281	7.7882
Our method ($w=1$, $\delta =0$)	0.0000	0.0000	7.0117
Our method ($w=1$, $\delta =1$)	0.0052	0.2160	7.8326
Our method ($w=1$, $\delta =2$)	0.0120	0.3550	7.8465
Our method ($w=1$, $\delta =5$)	0.0408	0.6525	7.8734
Our method ($w=2$, $\delta =1$)	0.0060	0.2326	7.8996
Our method ($w=2$, $\delta =2$)	0.0143	0.4110	7.9094
Our method ($w=2$, $\delta =5$)	0.0482	0.7735	7.9147

Bold indicates the best performance in each column

The images evaluated were all 8-bit; therefore, the maximum achievable entropy is 8

### Histogram equalization

The results of our histogram equalization experiments on the Flickr dataset are given in Table 2. Both the histogram deviation and entropy are reported in this experiment.

For this experiment, we measured the histogram deviation of the equalized image. We shall first provide the intuition behind this measure. Considering the goal of histogram equalization, using the naïve histogram equalization technique, the histogram deviation will measure zero error; however, the produced histogram is undesirably sparse. Conversely, [23] is capable of guaranteeing a near-perfectly flat histogram; however, the equalized intensity of pixels that originally shared the same value may now differ greatly. It is therefore logical to consider the problem of histogram equalization to be finding a suitable balance between these properties.

The method in [23] achieves the greatest entropy and demonstrates that it is possible to achieve a low mean intensity error while achieving maximal entropy on real images. The trade-off between entropy and intensity error is highlighted by the method’s maximum histogram deviation. The proposed method achieves a similar mean intensity error, while having a lower maximum deviation error.
